# Genome-Wide Screen for Differential DNA Methylation Associated with Neural Cell Differentiation in Mouse

**DOI:** 10.1371/journal.pone.0026002

**Published:** 2011-10-18

**Authors:** Rene Cortese, Jörn Lewin, Liselotte Bäckdahl, Manuel Krispin, Reinhold Wasserkort, Florian Eckhardt, Stephan Beck

**Affiliations:** 1 Epigenomics AG, Berlin, Germany; 2 University College London (UCL) Cancer Institute, University College London, London, United Kingdom; Bellvitge Biomedical Research Institute (IDIBELL), Spain

## Abstract

Cellular differentiation involves widespread epigenetic reprogramming, including modulation of DNA methylation patterns. Using Differential Methylation Hybridization (DMH) in combination with a custom DMH array containing 51,243 features covering more than 16,000 murine genes, we carried out a genome-wide screen for cell- and tissue-specific differentially methylated regions (tDMRs) in undifferentiated embryonic stem cells (ESCs), in *in-vitro* induced neural stem cells (NSCs) and 8 differentiated embryonic and adult tissues. Unsupervised clustering of the generated data showed distinct cell- and tissue-specific DNA methylation profiles, revealing 202 significant tDMRs (p<0.005) between ESCs and NSCs and a further 380 tDMRs (p<0.05) between NSCs/ESCs and embryonic brain tissue. We validated these tDMRs using direct bisulfite sequencing (DBS) and methylated DNA immunoprecipitation on chip (MeDIP-chip). Gene ontology (GO) analysis of the genes associated with these tDMRs showed significant (absolute Z score>1.96) enrichment for genes involved in neural differentiation, including, for example, *Jag1* and *Tcf4*. Our results provide robust evidence for the relevance of DNA methylation in early neural development and identify novel marker candidates for neural cell differentiation.

## Introduction

DNA methylation occurs predominantly as covalent modification of cytosines within a CpG sequence context. It represents the most stable epigenetic mark and has an impact on different biological processes in both healthy and diseased cells, including e.g. neural cell differentiation [1], [2], [3]. Large-scale DNA methylation profiling has demonstrated that tissue-specific differentially methylated regions (tDMRs) are highly correlated with cellular phenotypes [4], [5], [6]

Much effort has been made to understand the dynamics of DNA methylation during neural cell differentiation and the identification of epigenetic biomarkers that capture different aspects of cellular differentiation processes. ESCs are of particular interest in this context as they have previously been shown to acquire characteristic epigenetic marks during their differentiation from ESCs to NSCs and, subsequently, to tissues [7], [8]. Probably the best-known marker of neural cell differentiation is *Pou5f1* (usually referred as *Oct4*), encoding a homeobox protein essential in the maintenance of pluripotency [9]. A tDMR at this gene is hypomethylated in ESCs and hypermethylated in NSCs and terminally differentiated tissues [10]. In a recent targeted study, we reported a tDMR in the body of the *Ddah2* gene to be an epigenetic biomarker for neural stem cell differentiation [11]. Here, we conducted a genome-wide study to generate and analyze DNA methylation profiles of E.14 embryonic stem cells (ESCs) and *in vitro* induced neural stem cells (NSCs), as well as 4 embryonic and 4 adult murine tissues using a custom mouse DMH array.

Numerous methods have been developed for genome-wide DNA methylation profiling [12], [13]. Among them, Differential Methylation Hybridization (DMH) allows the detection of tDMRs by digesting genomic DNA into a defined fragment library using first methylation-insensitive restriction enzymes, adaptor ligation, digestion of unmethylated template fragments using methylation-sensitive restriction enzymes, adaptor-mediated amplification and subsequent hybridization to microarrays [14], [15]. By coupling this technology to custom-designed arrays, genome-wide coverage of DNA methylation profiles can be achieved [16]. Recently, we developed a mouse-specific DMH array that contains 51,243 features covering 17,384 genes and 16,656 promoter regions distributed across all chromosomes. Our results highlight the relevance of differential DNA methylation in neural cell differentiation and identify novel candidate markers for neural cell differentiation. Furthermore, as our data are compatible with a human-specific DMH array these results potentially enable an extrapolation to orthologous human genes.

## Results

### Tissue-specific DNA methylation profiles using DMH

We studied the DNA methylation profiles of E.14 ESCs and *in vitro* induced NSCs derived from this cell line [11], representing totipotent and pluripotent cellular development stages, respectively, 4 embryonic mouse tissues (limbs, spinal cord, forebrain and hindbrain) representing cells at a differentiated embryonal development stage, and 4 adult mouse tissues (spleen, liver, kidney and heart) representing terminally differentiated cells. In addition, we included enzymatically methylated (100% methylation) and unmethylated (0% methylation) control samples. These controls serve as calibrators for the quantification of the relative methylation value for each of the 51,243 features on the array. We generated and analyzed DNA methylation profiles using the DMH technology as described previously [15], but on a newly designed custom mouse array.

**Figure 1:**
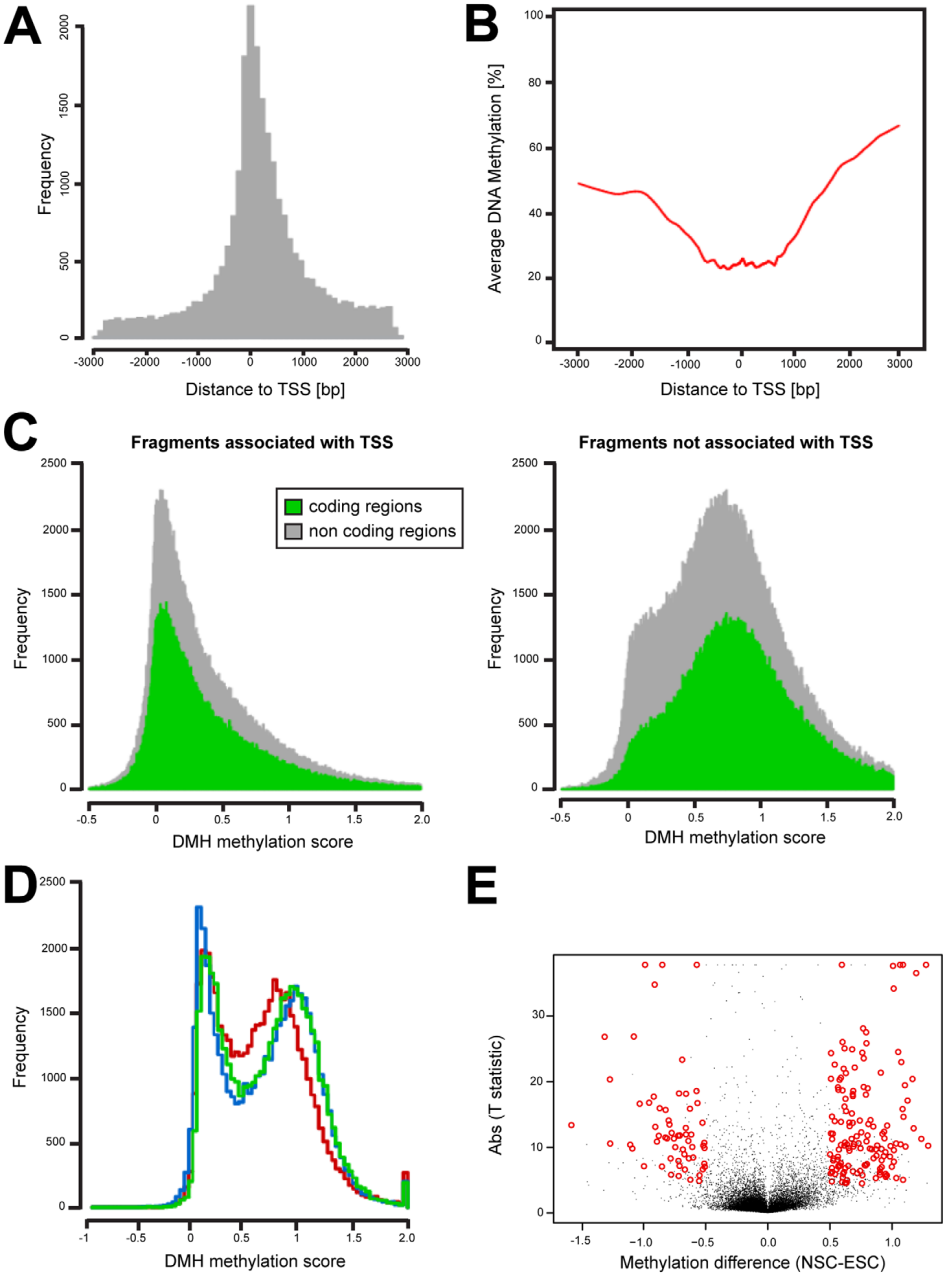
DNA methylation data distribution. A) DMH feature location across the genome. After array normalization and data processing, the full DMH dataset contained 51,243 features. Thereof, 23,957 features were associated with Transcriptional Start Sites (TSS) of annotated genes, 27,286 were located in distal CG rich areas. B) TSS-associated features (+/−1 kb range) are less methylated with respect to features not associated with TSS. The red line represents the average methylation percentages (Y-axis) in all tissues and cells across DMH features sorted by their distance to the TSS (X-axis). C) Methylation distribution was similar in coding and non-coding regions in features associated to TSS (left panel) and features not associated to TSS (right panel). Distribution of features located in coding and non-coding regions are represented as green and gray shapes, respectively. DMH scores represent the percentage of methylation calculated using enzymatically methylated (100% methylation) and unmethylated (0% methylation) control samples as calibrators. Thus, DMH scores range from 0 to 1, representing 0% and 100% methylation, respectively D) ESCs, NSCs and embryonic brain samples showed bimodal distribution with peaks at 0 (0% methylation) and 1 (100% methylation). While ESCs and NSCs displayed similar distribution with well-differentiated peaks, embryonic brain distribution is shifted towards more intermediate values. Distribution of DMH scores in NSCs, ESCs and embryonic brain are represented as blue, green and red lines, respectively. E) Volcano plot of features showing differential methylation in ESCs and NSCs. 140 regions were highly methylated in NSCs and 62 highly methylated in ESCs. Features were ranked and candidate tDMRs were selected following the criteria detailed in the text. X-axis represents the methylation percentage difference between ESCs and NSCs while Y-axis represents the registered t-statistic for that difference. Red circles highlight candidate tDMRs. Features showing higher methylation in NSCs and ESCs are clustered on the right and left sides, respectively.

To explore the DNA methylation distribution across the murine genome, we studied the relationship between the feature location and methylation content in all samples (Figure 1). After array normalization and data processing, the full DMH dataset contained 51,243 features. Of these, 23,957 features were associated with Transcription Start Sites (TSS) of annotated genes and 27,286 were located in distal CpG rich areas (Figure 1A). The average methylation values of the features located within a range of 1000 bases upstream or downstream to the TSS were low (20% average methylation). Features located outside this range showed increasing methylation values towards hypermethylation (Figure 1B). The majority of TSS-associated features had low methylation scores (<10%) while features not associated with TSS showed higher values towards hypermethylation (>75%). We did not detect differences in the average methylation between the samples studied when the features were located in non-coding (i.e. promoter) or coding (i.e. exon 1 or intron 1) regions, regardless of their association with TSS (Figure 1C). To study the differences in DNA methylation distribution in individual tissues and cells, we compared the methylation percentage distribution in ESCs, NSCs and embryonic brain (Figure 1D). DMH scores in these samples have a bimodal distribution, with one peak corresponding to unmethylated features (0% methylation) and the other peak corresponding to hypermethylated features (100% methylation). While ESCs and NSCs displayed similar distributions with well-differentiated peaks, the distribution in embryonic brain is shifted towards more intermediate values.

### DNA methylation profiles of ESCs, NSCs and embryonic brain

We studied the variation of DNA methylation profiles among ESCs, NSCs and embryonic brain and analyzed the data to identify candidate markers which potentially play a role during neural cell differentiation. First, we separated the features on the DMH array according to their association to the TSS. Hence, we obtained two independent datasets that can be interpreted independently. In this study, we focused on TSS-associated features. Similar analyses were also performed on non TSS-associated features (data not shown).

**Figure 2:**
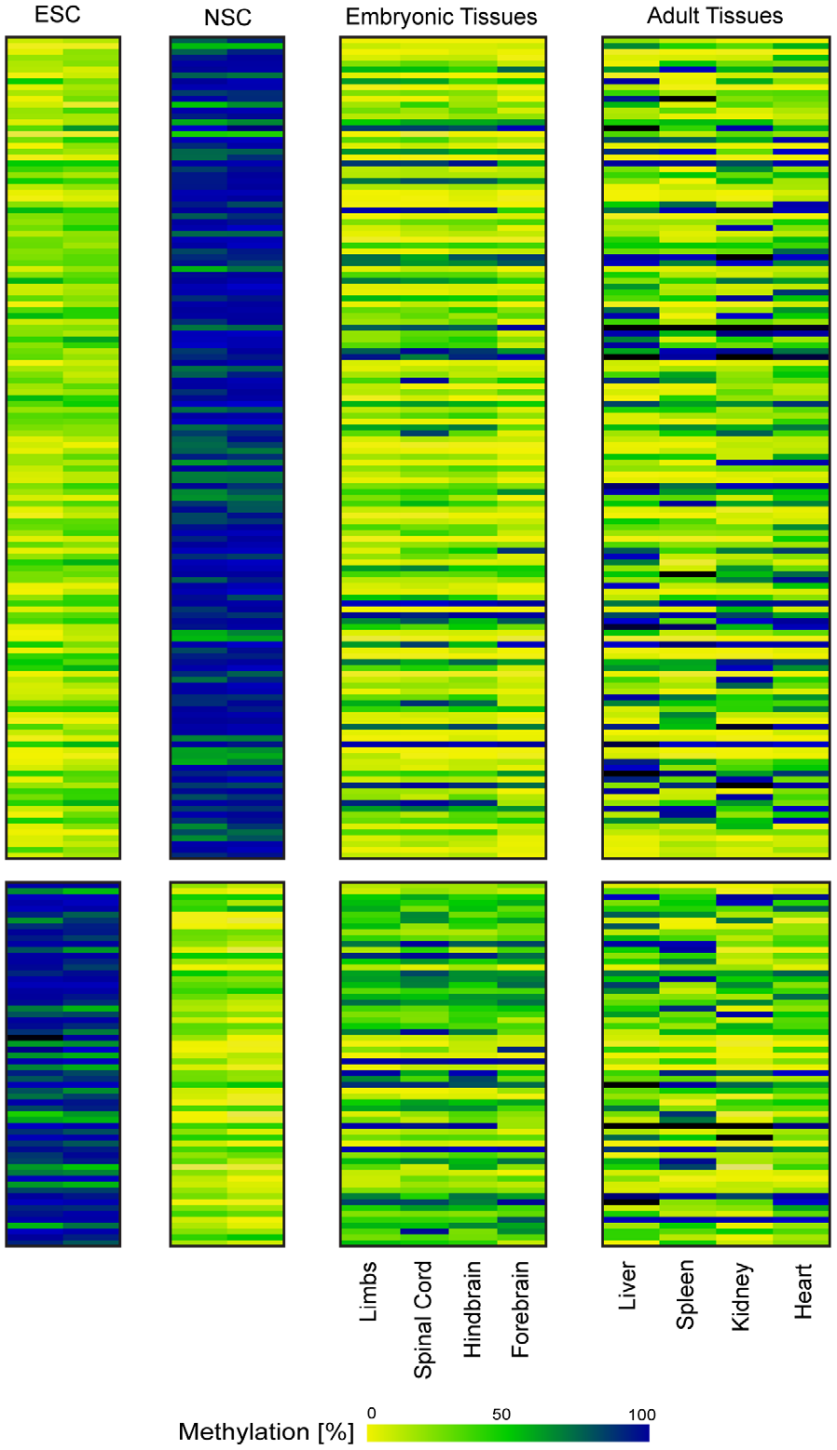
TSS-associated tissue-specific differentially methylated regions (tDMRs) in stem cells and embryonic tissues. 202 candidate tDMRs were discovered comparing profiles in NSCs and ESCs. Each row corresponds to a feature in the DMH array while each column corresponds to a sample, i.e. NSCs (n = 2), ESCs (n = 2) embryonic and adult tissues (n = 8). Quantitative methylation analysis results are shown in a color code ranging from yellow (∼0% methylation), over green (∼50% methylation) to dark blue (∼100% methylation).

Figure 1E shows a volcano plot identifying differentially methylated regions between NSCs and ESCs. We ranked all features according to the effect size of the differential methylation (M) and the T- statistic (T) values and selected candidate tDMRs. The complete list of tDMRs and their associated genes are provided in the supplementary information (Supplementary Table S1). Out of the 202 top-ranked tDMRs, 140 displayed higher methylation in NSCs versus ESCs while 62 displayed the opposite pattern, i.e. higher methylation in ESCs and lower in NSCs (Figure 2).

**Figure 3:**
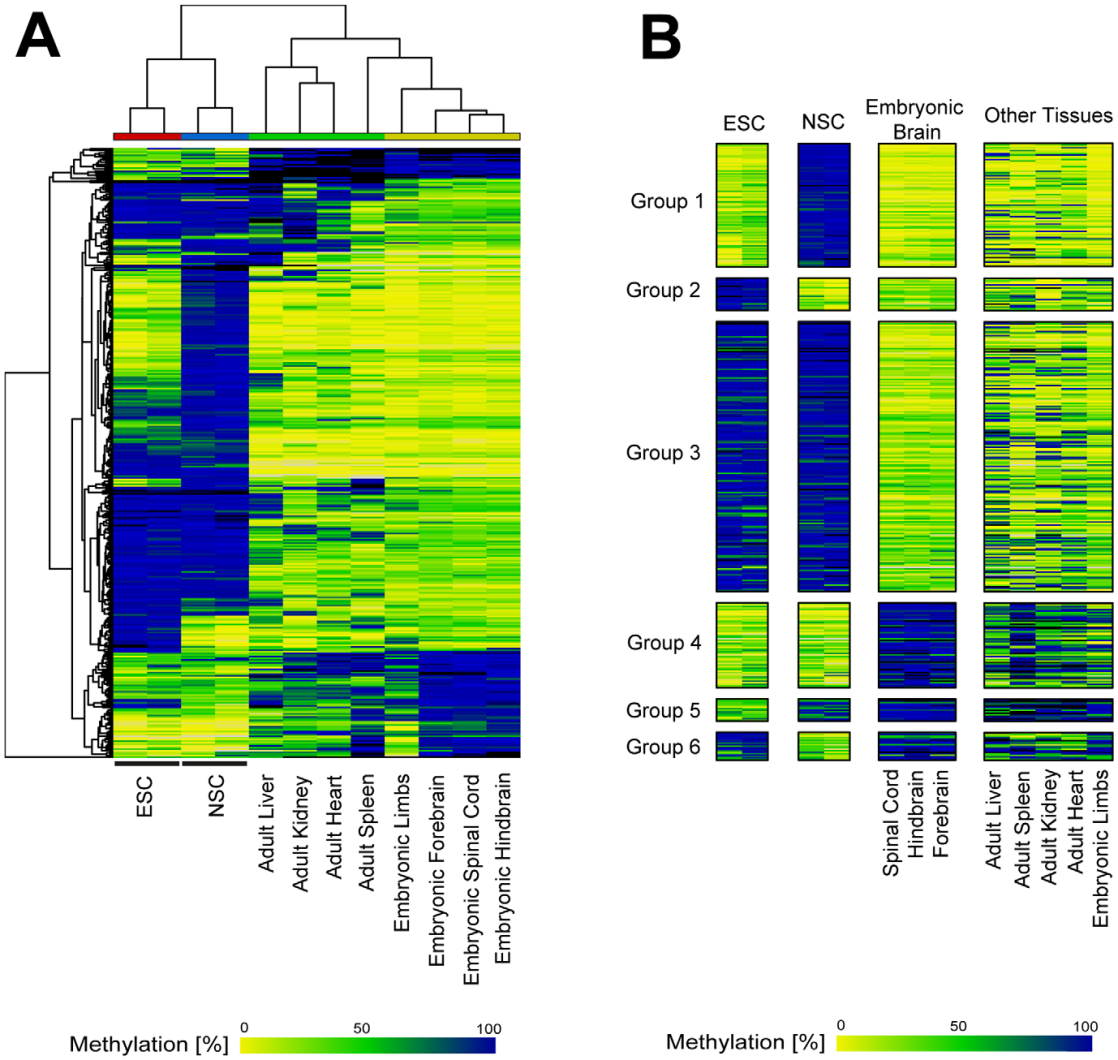
TSS-associated tDMRs define distinct groups in NSCs, ESCs and embryonic brain. A) Unsupervised clustering of top-ranked candidates. B) ANOVA of methylation percentage in ESCs, NSCs and embryonic brain defined 6 tDMR groups. 382 candidate tDMRs were selected and ranked. Color code as detailed in Figure 2.

Next, we studied if the identified tDMRs were related to the degree of neural differentiation. We selected 382 candidate tDMRs according to the following criteria: p-value lower than 0.05, effect size higher than 0.5 and minimum DMH score higher than 0.5 in at least one group. Unsupervised clustering of these candidates revealed distinct groups among NSCs, ESCs and embryonic brain samples (Figure 3A). Variance analysis (ANOVA) of the methylation percentage defined 6 tDMR groups (Figure 3B). For example, group 1 contained 84 tDMRs which were highly methylated in NSCs but unmethylated in ESCs and embryonic brain. Among the genes associated with the tDMRs in group 1, we found genes related to stem cell differentiation (*Sox10*) and cell proliferation (*Lhx9*, *Gbx2*, *Emx2*), as well as genes related to the development of the brain and the neural system (*Gbx2*, *Tbr1*, *Slit2*, *Sema6a*). The complete list of tDMRs in each group and their associated genes are provided in supplementary table S2.

Taken together, our data strongly suggest that DNA methylation may play a major role during murine neural stem cell differentiation. To explore whether the genes associated with tDMRs were functionally related, we used the MAPPFinder software [17] to assess the Gene Ontology (GO) groups overrepresented among them. Supplementary table S3 contains the MAPPFinder results for these tDMR-associated genes ranked by their corresponding Z-score. Groups over-represented in genes associated with tDMRs that were highly methylated in ESCs, were mostly related to brain and central nervous system development (e.g. “nervous system development”, GO ID: 7399, 7/15 included genes, Z-score: 1.945) and regulation of gene expression (e.g. “transcription”, GO ID: 6350, 10/23 included genes, Z-score: 2.884). Conversely, many groups over-represented in genes associated with tDMRs that were highly methylated in NSCs, were related to membrane and lipid metabolism (i.e. “cellular lipid metabolic process”, GO ID: 44255, 5/5 genes changed, Z-score: 1.546) and phosphatase activities (e.g. “nucleoside-triphosphatase activity”, GO ID: 17111, 5/5 genes included, Z-score: 1.546).

### Validation of candidate tDMRs using direct bisulfite sequencing

To validate the tDMRs identified by murine-specific DMH with a different technology, we used direct bisulfite sequencing (DBS) [4], [18], [19]. We selected 51 tDMRs (see Supplementary Table S2), choosing predominantly candidates with large effect sizes in the ANOVA analysis and including candidates that were either hypermethylated or hypomethylated in ESCs.

**Figure 4:**
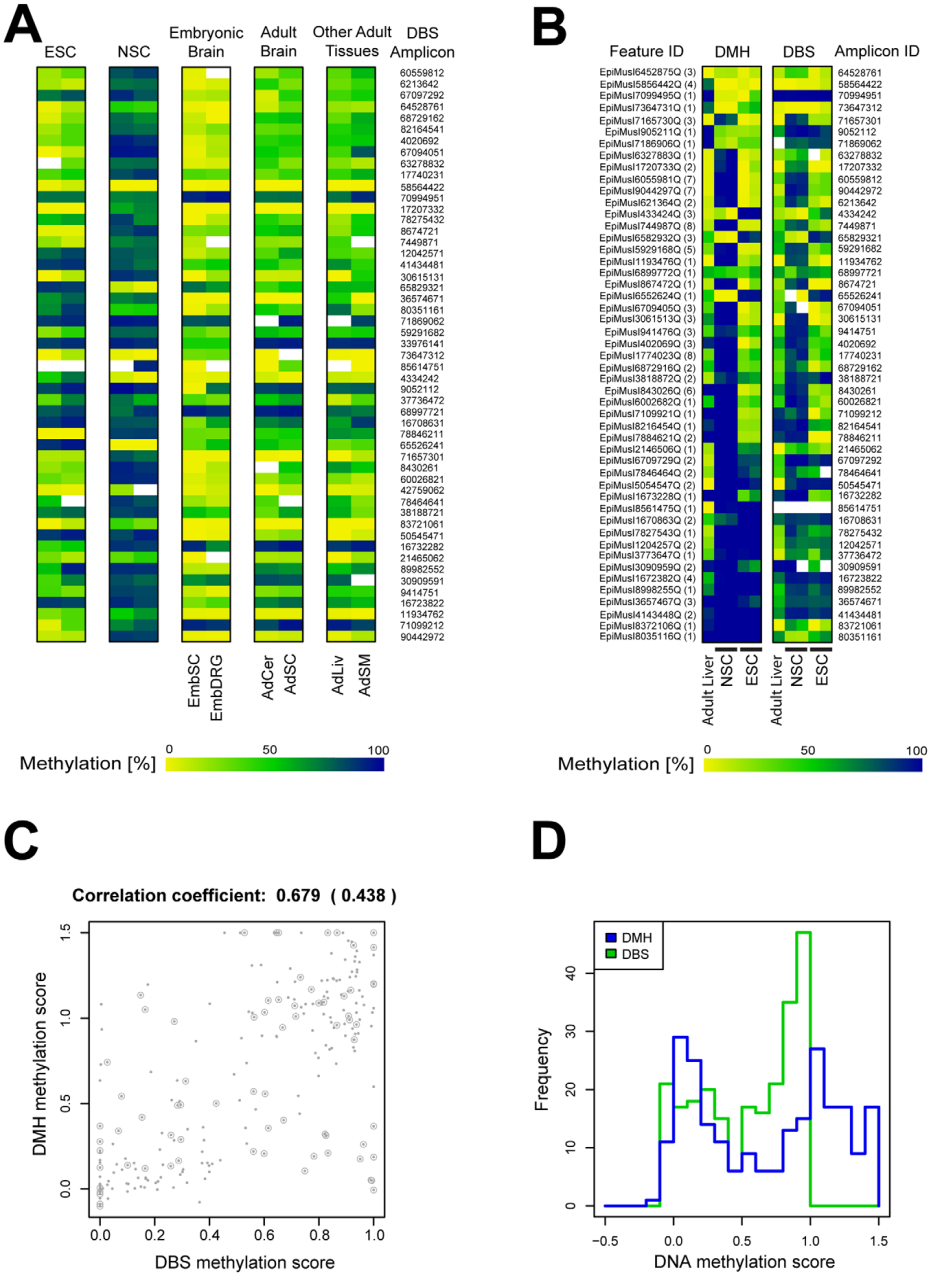
tDMR validation by direct bisulfite sequencing. A) Averaged methylation values obtained by direct bisulfite sequencing (DBS). 51 candidate regions were studied in embryonic stem cells (ESCs), neural stem cells (NSCs), embryonic spinal cord (EmbSC), embryonic dorsal root ganglia (EmbDRG), adult cerebellum (AdCer), adult spinal cord (AdSC), adult liver (AdLiv) and adult skeletal muscle (AdSM). Color code as detailed in Figure 2. Rows represent each DBS amplicon and columns correspond to the average methylation value per sample type. B) DMH methylation percentage and averaged DBS amplicon methylation values in NSCs and ESCs. 49 matched DMH features/DBS amplicons were studied in the same biological samples. Data are presented in two color-matching matrices for DMH and DBS (left and right matrix, respectively). The numbers in brackets next to each feature ID indicate the number of restriction sites in the respective DMH fragments. Rows represent each DMH feature or DBS amplicon and columns correspond to the average methylation value per sample type. Color code as detailed in Figure 2. C) Correlation analysis of DMH and DBS data. Mean CpG methylation obtained by DBS are shown on the X-axis and DMH scores are showed on the Y-axis. The correlation coefficient was 0.679 for all DMH features/DBS amplicon pairs, while 0.438 for pairs for which the DMH feature contained a single restriction site. Points in circles highlight features with a single restriction site. D) DNA methylation data distribution in DMH features and DBS amplicons. Data distribution was similar with both technologies. The blue line represents the distribution of the DMH scores while the green line the distribution of mean DNA methylation for DBS amplicons.

DBS validation of these 51 tDMRs was done on amplicons from five different biological samples: ESCs, NSCs, embryonic neural tissue (spinal cord and dorsal root ganglia), adult neural tissue (cerebellum and spinal cord) and adult non-neural tissues (liver and skeletal muscle) (Figure 4A). To evaluate the correlation between DMH and DBS quantitatively, we compared the averaged methylation values obtained by DMH and DBS on the same biological samples using ESC, NSC and adult liver (Figures 4B, C and D). The correlation coefficient was 0.679 when all DMH features/DBS amplicon pairs were considered. However, for DMH features containing only a single restriction site the correlation coefficient decreased to 0.438 (Figure 4C). The observed methylation data distribution was comparable with both technologies (Figure 4D) showing a bimodal distribution with peaks for unmethylated (0% methylation) and for fully methylated (100% methylation) amplicons or DMH features, respectively.

### Correlation between DMH and MeDIP data

In a previous study, we used an affinity capture method (MeDIP-chip) to screen mouse chromosome 17 for neural differentiation biomarkers in ESCs and NSCs [11]. Taking advantage of the fact that MeDIP-chip and DMH profiling was performed on the same biological samples; we compared the methylation values in regions of particular interest obtained with both technologies.

**Figure 5:**
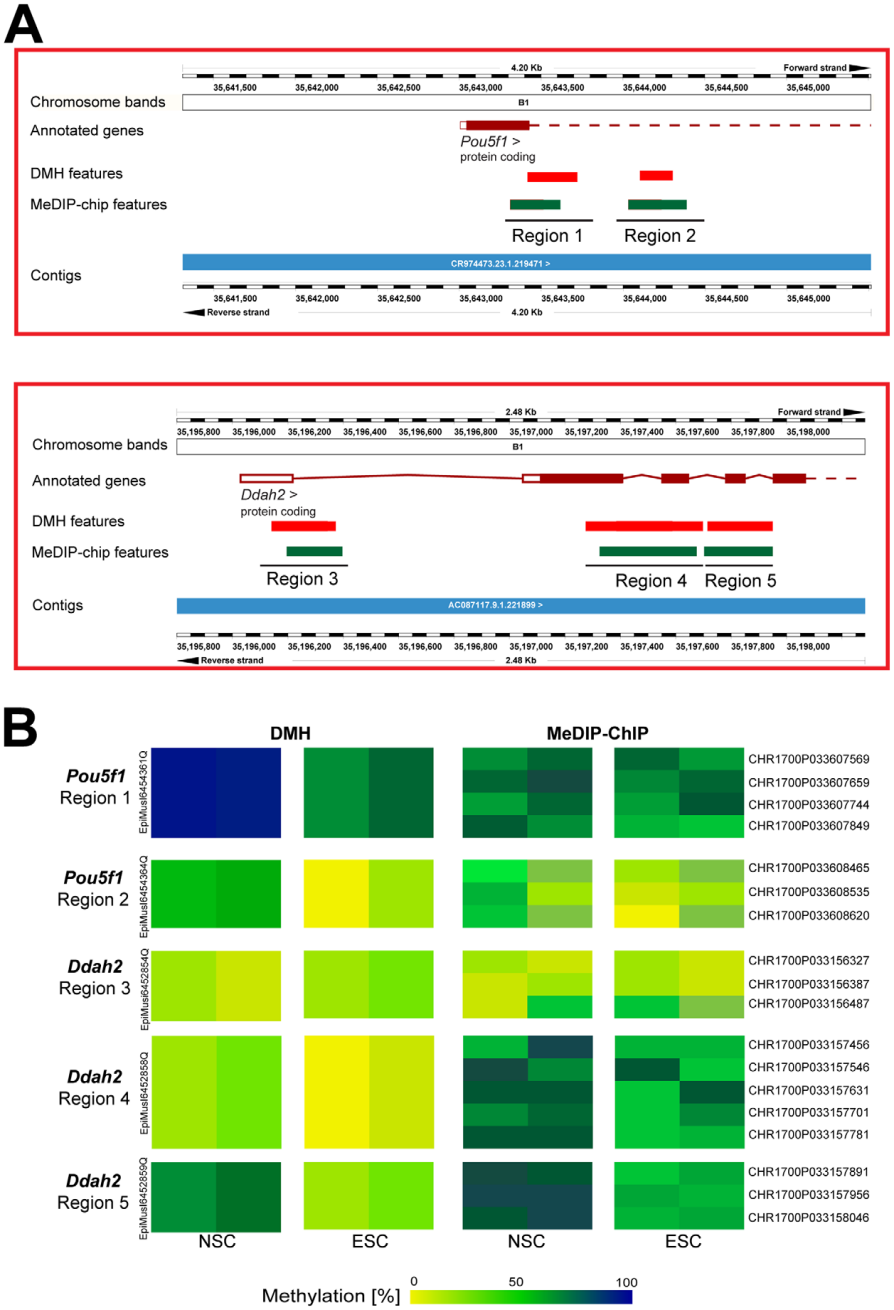
DMH and MeDIP-chip DNA methylation profiles in NSCs and ESCs. Both technologies showed similar results for two genes involved in neural differentiation. A) Location of probes covering the investigated regions in the *Pou5f1* (upper panel) and *Ddah2* (lower panel) genes. Red and green boxes represent areas covered by features in the DMH and MeDIP-chip arrays, respectively. B) DNA methylation values in *Pou5f1* and *Ddah2* genes in NSCs and ESCs. DNA methylation values obtained with DMH (left panel) and MeDIP-chip (red panel) are comparable. Rows correspond to features in the DMH or MeDIP-chip array and columns correspond to samples (NSCs (n = 2) and ESCs (n = 2)), grouped for DMH and MeDIP-chip respectively. Quantitative methylation values are expressed as methylation percent for DMH and MeDIP-chip respectively and color-coded as in Figure 2.

Of the 202 genes associated with the top-ranking tDMRs from the DMH analysis of NSCs and ESCs, 8 were also included on the MeDIP array (Table 1). Of these, 6 genes (*Pou5f1*, *Ddah2*, *Nr4a2*, *Itpka*, *Btbd17* and *Emx2*) were hypermethylated in NSCs, while 2 genes (*Fam179a* and *Fbxl17*) were hypermethylated in ESCs. We focused our analysis on two genes that have been reported as epigenetic biomarkers for neural stem cell differentiation: *Pou5f1* [20] and *Ddah2* [11]. Figure 5A shows the location of the features covering the investigated regions on the DMH (red boxes) and MeDIP (green boxes) arrays for *Pou5f1* (upper panel) and *Ddah2* (lower panel), respectively. For the *Pou5f1* gene, we studied two regions covering the exon 1-intron 1 junction (Region 1) and intron 1 (Region 2), respectively. We studied 3 regions for the *Ddah2* gene, Region 3 covering the exon 1-intron 1 junction and Regions 4 and 5 located within the gene body. In general, the results obtained by DMH and MeDIP-chip were comparable for these regions. For example, we detected differential methylation in Region 2 by both methods, DMH and MeDIP (Table 1 and Figure 5B). This region was intermediately methylated in ESCs (65% and 38%, for DMH and MeDIP, respectively), while highly methylated in NSCs (91% and 42%). Likewise, Region 2 was lower methylated in ESCs (4% and 3%) than in NSCs (45% and 19%). We detected differential methylation only in the gene body of the *Ddah2* gene but not at the 5′ end of the gene (Figure 5B, lower panel) by both methods. At the 5′ end of the gene, Region 3 showed lower methylation in both, ESCs (11% and 10%) and NSCs (14% and 13%). In the gene body, however, Regions 4 and 5 showed higher methylation in NSCs than in ESCs. Region 4 was unmethylated in ESCs (3% and 34%) while moderately methylated in NSCs (21% and 47%). Region 5 was moderately methylated in ESCs (21% and 23%), while intermediately methylated in NSCs (60% and 67%). However, we detected differential methylation in Region 1 within the *Pou5f1* gene only by DMH.

**Table 1:** DNA methylation values assessed by DMH and MeDIP-chip.

DMH methylation				MeDIP-Chip methylation		
Associated gene	DMH feature	NSCs (%)	ESCs (%)	MeDIP probeset	NSCs (%)	ESCs (%)
***Pou5f1 (Oct 4)*** ** – Region 1**	EpiMusI6454361Q	91	65	CHR1700P033607569	39	41
				CHR1700P033607659	39	41
				CHR1700P033607744	43	39
				CHR1700P033607849	46	34
***Pou5f1 (Oct 4)*** ** – Region 2**	EpiMusI6454364Q	45	4	CHR1700P033608465	22	3
				CHR1700P033608535	22	3
				CHR1700P033608620	14	4
***Ddah2*** ** - Region 3**	EpiMusi6452854Q	11	14	CHR1700P033156327	8	9
				CHR1700P033156387	8	9
				CHR1700P033156487	16	22
***Ddah2*** ** - Region 4**	EpiMusI6452858Q	21	3	CHR1700P033157456	17	22
				CHR1700P033157546	25	29
				CHR1700P033157631	56	44
				CHR1700P033157701	63	45
				CHR1700P033157781	75	31
***Ddah2*** ** - Region 5**	EpiMusI6452859Q	60	21	CHR1700P033157891	73	25
				CHR1700P033157956	65	23
				CHR1700P033158046	65	23
***Nr4a2***	EpiMusI621364Q	100	11	CHR200P057112628	67	61
				CHR200P057112693	44	42
				CHR200P057112803	73	66
***Itpka***	EpiMusI843026Q	98	29	CHR200P119484622	55	32
				CHR200P119484712	52	31
				CHR200P119484777	49	29
***Btbd17***	EpiMusI9044297Q	100	11	CHR1100P114900476	78	24
				CHR1100P114900556	35	46
				CHR1100P114900656	24	44
				CHR1100P114900731	35	22
***Fbxl17***	EpiMusI6552624Q	7	100	CHR1700P061493554	21	93
				CHR1700P061493659	34	92
				CHR1700P061493719	38	90
***Fam179a***	EpiMusI6582932Q	1	70	CHR1700P069972763	23	61
				CHR1700P069972863	15	58
				CHR1700P069972963	13	55
***Emx2***	EpiMusI7165730Q	80	10	CHR1900P059382729	27	10

## Discussion

### DNA methylation profiles obtained by DMH allow differentiation of tissue and cell types in mouse

The high genomic coverage of our DMH microarray platform enabled the evaluation of genome-wide profiles, showing characteristic features of DNA methylation across the murine genome in tissues and cell lines. In general, the profiles showed the classical bimodal distribution with peaks at 0% and 100% methylation relative to methylated control DNA, respectively. However, tissues showed an increased number of intermediate DNA methylation values. As tissues have a more heterogeneous cellular composition than cell cultures, DNA methylation values will correspond to the average values across different cell types and their degree of differentiation. In addition, we observed mainly hypomethylation of regions neighbouring the TSS and increased methylation content in distal regions of coding and non-coding regions. This is in line with the distribution of methylation observed in human studies [4], [21], [22]. In a previous work, we reported a high correlation between the tissue specific differential methylation in human and mouse [4]. As the murine DMH microarray contains orthologous regions to those contained in our human DMH microarray, results can potentially be extrapolated to similar studies using human tissue and cell samples. The corresponding annotation between orthologous genes enables comparative genome-wide epigenetic profiling in animal models; as would be required, for example, in the pre-clinical evaluation of drug effects and toxicity. Furthermore, the DMH profiles of terminally differentiated mouse tissues presented in this study represent a comprehensive and valuable dataset which will be subjected to more detailed investigation of tissue-specific DNA methylation differences in a future study.

Although we can not rule out the possibility that some of the observed differences between ESCs and NSCs and adult and embryonic tissues are due to different genetic backgrounds, our previous findings suggest that specific DNA methylation differences in non-malignant tissues are more frequent and pronounced than methylation differences due to inter-individual differences [4]. In this regard, studies comparing inbreed mice generally focus on phenotypical traits that can be attributed to genetic differences [23], [24], rather than the study of overall variation in DNA methylation profiles in similar tissues of different mice strains. Schilling and colleagues reported 435 regions associated to 171 genes showing strain-specific DNA methylation, when DNA methylation profiles of macrophages from C57BL/6 and BALB/c were compared [25]. However, it should be considered that the studied regions in that work were selected from differential expression patterns that might explain the immunological differences between these two strains and therefore, extrapolation to the whole methylome might result in an overestimation of the variation of the DNA methylation profiles. The extent of epigenetic variation that can be attributed to genetic differences have been studied in humans using monozygotic and dizygotic twins. Kaminsky and colleagues showed that matched monozygotic twins had significantly higher intraclass correlations than dizygotic twins [26]. In the same study, they did not detect significant variation in the distribution of DNA methylation variation between inbred and outbreed mice at 2,176 unique genomic regions using a methylation-sensitive enzymatic restriction strategy coupled to microarray. Taken together these findings support the idea of epigenetic variation originating at the zygote stage, without being limited to DNA sequence variation. In order to reach more comprehensive insights, a combined study using unbiased and high-coverage approaches for sequencing (i.e. deep sequencing) and DNA methylation profiling (i.e. DMH or MeDIP) in non-malignant tissues of different mouse strains will be required.

The comparison of DMH and DBS data for differentially methylated regions resulted in a correlation of 68%. This correlation, however, decreased to 44% when the DMH features contained only a single DNA methylation-sensitive restriction site. In DBS amplicons corresponding to DMH fragments with a single restriction site, only the methylation value for the CpG within the site is considered. When DBS amplicons corresponded to DMH fragments containing multiple restriction sites, methylation values were averaged for the CpGs included in those sites.. Therefore, we expect that fragments with single restriction sites may be more sensitive to variability, due to intrinsic sample factors, technical performance or other experimental parameters. As the proportion of fragments containing single and multiple restriction sites fragments in our DBS analysis (19 vs. 29; Figure 4B) is similar to the overall representation of these fragments on the DMH array, differences in the correlation coefficients between fragments with single and multiple restriction sites are probably not caused by biases in the number of fragments containing single or multiple restriction sites. The lower correlation coefficient does not diminish, however, the concordance rate in an important number of new marker candidates, as can be seen in Figure 4D and table S1. Likewise, when we compared the results from our previous work with these cells [11], we found concordant results between mouse DMH and MeDIP not only for the neural differentiation markers, *Pou5f1* and *Ddah2*, but also for other loci that were covered by both arrays. Since these technologies use different approaches for methylation detection (restriction enzymes, sodium bisulfite conversion and antibody against 5-MeC, for DMH, DBS and MeDIP respectively), the concordance highlights the strength of the findings. However intrinsic technical limitations as discussed by Irizarry and colleagues [27] cannot be excluded.

For many features we observed DMH scores higher than 1 (representing 100% methylation, as extrapolated by methylated control sample signals). There are three possible major sources for such measurements. 1) The variance around 100% is much larger than in the lower methylation range. As DMH utilizes a genome-wide PCR, it might lead to broader peaks at the fully methylated side of the mainly bimodal methylation distribution and thereby, extending the extrapolated rates beyond 100%. 2) The enzymatically methylated reference DNA might partially be incompletely methylated, which would lead to an over-estimation at the affected sites. 3) Since DHM assesses the total amount of methylated DNA, an increase in the copy number of a methylated region will also lead to an increased amount of detected DNA and therefore to a significantly higher methylation percentage. Thus, tissue- specific copy number increases with respect to the 100% calibrator DNA (hypermethylated control, defined as 100% methylation) will result in scores higher around than 1.5 for copies in triplicate and beyond for sites with multiple copies. Thereby, it is very likely that linear interpolation to estimate the exact copy number is not possible with data from the DMH array used in this study. Interestingly scores beyond 1.5 seen in cancer tissue and cancer cell line DMH experiments have been used successfully to obtain consistent information about regions with copy number effects, especially if such measurements were observed at multiple neighboring sites (unpublished data). Therefore it might also be of interest to investigate DMH scores larger than 1.5 as found in the mouse cell lines used in this study.

### Comparison of ESCs, NSCs and embryonic brain identifies differentially methylated genes during neural differentiation

Many of the identified tDMRs may represent novel epigenetic markers for neural cell differentiation. Among the genes associated with tDMRs between ESCs and NSCs, many have a known function in neural differentiation (i.e. *Jag1*, *Tcf4*) or have a function related to the neural system (i.e. *Mtap2*, *Slitrk1*), which likely supports a major role for DNA methylation in neural stem cell differentiation that was unknown in most cases.

The genome-wide nature of our DMH analysis allowed not only discovery of individual candidate markers, but also of groups of genes which presumably act coordinately, and which were characterized by functional epigenetic signatures defining a particular differentiation stage. For example, genes associated with tDMRs in NSCs and ESCs shared common GO annotations (Supplementary Table S3). GO groups overrepresented in genes highly methylated in ESCs, and therefore probably silenced, were mainly related to brain and neural system development (e.g. GOID 7399 and GOID 7420). These findings support our hypothesis of DNA methylation playing a major functional role in neural development, acting on individual genes and molecular pathways. Interestingly, GO groups overrepresented in genes highly methylated in NSCs were related to lipid metabolic process (GOID 6629) and nucleoside-triphosphatase activity (GOID 17111). It has been suggested that the interplay between metabolites of glycerophospholipid and sphingolipid metabolism may play an important role in neural cell differentiation [28]. Our data suggest that this interplay may be regulated through differential DNA methylation.

Taken together, our findings emphasize the functional nature of DNA methylation during cell differentiation and, more specifically, in neural cell differentiation. The discovery of novel tDMRs during differentiation from pluripotent embryonic stem cells to terminally differentiated tissues provides attractive candidates for more detailed functional studies of key drivers of neural cell development.

## Materials and Methods

### Ethical statement

Adult and embryo mice tissues were kindly provided by Dr. Edith Heard (CNRS Institute Curie, Paris). Husbandry, housing and experiments of animals comply with the French legislation and the European regulations for the Protection of Vertebrate Animals used for Experimental and other Scientific Purposes. The institution is approved to carry out animal work and the personnel handling the animals have been trained and licensed accordingly. All the work with animals has been done at the Specified Pathogen Free central mouse facility at the Institut Curie. This facility has been accredited by the French National Authority (Accreditation number #B75-05-18). Further ethical approval was not necessary as the study did not require in-vivo experimentation, but the molecular study of tissues obtained from sacrificed animals and established murine cell lines.

### Samples

#### Mouse tissue and DNA isolation

Adult mice were euthanized and organs (spleen, kidney, liver and heart) were dissected. Embryonic mice were 13.5 days old and 4 tissues were dissected (limbs, spinal cord, forebrain and hindbrain). All adult and embryonic tissues were obtained from F1 (C57BL/6×DBA/2J) mice. Adult mice DNA samples were taken from one mouse, while embryonic DNA corresponds to pools of 5 to 8 embryos. DNA was isolated from embryonic and adult mouse tissue using the MinElute kit (Qiagen, Hilden, Germany).

#### Cell culture, in vitro differentiation

For the analysis of ESCs, we used an already established murine cell line (E14) derived from C57BL/6 mice [29]. Cell culture, differentiation to NSCs and DNA isolation were performed as previously described [11].

### Differential Methylation Hybridization – DMH

DNA from each biological sample was split into two aliquots and both aliquots were processed in parallel. These two technical replicates were then hybridized to two arrays. Each pair of replicates showed a high correlation (mean R-squared value = 0.9729+/−0.0136). DMH scores used for further analysis were calculated using the averaged signals of the two technical replicates. DNA methylation profiles in NSC and ESC were assessed in two independent cultures for each cell type. The embryonic tissue group consisted of limbs (n = 1), spinal cord (n = 1), hindbrain (n = 2) and forebrain (n = 1). The adult tissues group consisted of 4 different tissues: liver (n = 1), spleen (n = 1), kidney (n = 1) and heart (n = 1). Pairs of biological replicates, when available, also showed high correlation in their respective DMH scores (R-squared values = 0.8955, 0.7903 and 0.8852 for NSC, ESC and hindbrain, respectively).

Hypermethylated regions were enriched using DMH as originally described by Huang et al. [14] and further optimized for the hybridization to high coverage Affymetrix human custom arrays [15], [16]. Briefly, genomic DNA was fragmented using a cocktail of DNA methylation- insensitive restriction enzymes (*Csp6I*, *MseI* and *BfaI*) and adaptors were ligated to the generated ends. The adaptors were prepared by annealing the two oligos (5′-AGGCAACTGTGCTATCCGAGGGAT-3′ and 5′-TAATCCCTCGGA-3′). Next, fragments were digested using a cocktail of DNA methylation-sensitive restriction enzymes (*BstUI*, *HpaII*, *HinP1I* and *HpyCH4IV*), which cleave unmethylated sites while leaving methylated sites intact such that subsequent PCR with primers complementary to the adaptors amplifies only the intact fragments. PCR products are then hybridized to microarrays.

In this work, we used a newly developed Affymetrix mouse array. This array was custom-designed (Affymetrix CustomSeq®) and contained 51,243 probesets, with each probeset consisting of 8–10 individual probes. The mouse DMH array was designed containing orthologous regions to the human DMH array in order to enable also human-mouse comparative studies. The probesets cover CpG-rich loci in 5′-untranslated regions, exons and introns of known genes, as well as in intergenic regions, across all murine chromosomes. Approximately 50% of the probes are located in know promoter regions. Furthermore, the array contains probesets for loci that do not contain any relevant methylation-sensitive restriction sites serving as internal calibration controls in addition to Affymetrix-designed control oligos. Data were analyzed according to the methods previously described [15], [16]. All microarray data is MIAME compliant and it has been deposited in the ArrayExpress database (Accession Number: E-MTAB-576)

### Direct Bisulfite Sequencing – DBS

Genomic DNA was bisulfite converted and PCR amplified as previously described [4]. Bisulfite-specific primers with a minimum length of 18 bp were designed using a modified Primer3 program [30]. Primer sequences are given in Supplementary Table S4. The target sequence of the designed primers contained no CpGs allowing an unbiased amplification of both unmethylated and methylated DNAs. Primers were also tested for specificity by electronic PCR (ePCR) [31]. PCR amplicons from bisulfite treated products were quality controlled by agarose gel electrophoresis, purified with ExoSAP-IT (USB Corporation, Cleveland OH, USA) to remove any excess nucleotides and primers, and sequenced directly in forward and reverse directions. For sequencing, we used the same primers as in the amplification reaction. Sequencing was performed on an ABI 3730 capillary sequencer using 1/20th dilution of ABI Prism BigDye terminator V3.1 sequencing chemistry. The PCR amplification profile was: Hotstart at 96°C for 30 seconds followed by 44 cycles of 92°C for 5 seconds, 50°C for 5 seconds and 60°C for 120 seconds. Before injection, products were purified on DyeEx plates (Qiagen, Hilden, Germany). The obtained sequencing chromatograms were used to quantify the methylation at given CpGs as previously described [4], [18], [19].

For each selected candidate tDMR, 2 amplicons were designed and each covered at least one restriction site contained in the corresponding DMH feature. The quality of the amplicons was evaluated by assessing the quality of the bisulfite sequencing reads first on technical DNA and the completeness of amplicon coverage. The better performing amplicon for each tDMR was then selected for the panel of amplicons for the validation analysis.

### Methylated DNA Immunoprecipitation and array hybridization – MeDIP-chip

Methylated DNA Immunoprecipitation was performed of DNA isolated from ESCs and NSCs according to standard protocols [32]. For the array analysis, we used a custom tiling array (NimbleGen) of mouse chromosome 17. The array comprised ∼385,000 isothermal probes with an average size of 50 bases, tiled at 100 base pair intervals. The tiling path was constructed for 2 kb windows that contained less than 20% repeat elements and at least 1% CpG density. The microarrays were processed and analyzed as described previously [33].

### MAPPFinder

MAPPFinder was used to correlate microarray data of differentially expressed genes to pathways annotated in GenMAPP and to Gene Ontology (GO) annotations [17]. This software calculates a cumulative total of genes changed for a pathway or GO group and assigns a statistical value, the Z-score.

## Supporting Information

Table S1 **tDMRs and their associated genes.**
(XLS)

Table S2 **tDMR-associated genes in ESCs, NSCs, and embryonic brain.**
(XLS)

Table S3 **MAPPFinder results for over-represented GO groups in ESC and NSC tDMRs.**
(XLS)

Table S4 **Primer sequences for direct bisulfite sequencing (DBS).**
(XLS)
